# Use of bone‐modifying agents and clinical outcomes in older adults with multiple myeloma

**DOI:** 10.1002/cam4.2591

**Published:** 2019-09-30

**Authors:** Adam J. Olszewski, Peter M. Barth, John L. Reagan

**Affiliations:** ^1^ Alpert Medical School of Brown University Providence RI USA; ^2^ Division of Hematology‐Oncology Lifespan Cancer Institute Providence RI USA

**Keywords:** bisphosphonates, bone‐modifying agents, health services research, multiple myeloma, SEER‐Medicare, supportive care, zoledronate

## Abstract

**Background:**

Guidelines recommend bone‐modifying agents (BMAs) for all patients initiating treatment for myeloma. We examined adherence to this recommendation, and BMA effectiveness in the era of bortezomib/lenalidomide‐based therapy among Medicare beneficiaries.

**Methods:**

From the linked Surveillance, Epidemiology, and End Results‐Medicare registry, we selected beneficiaries receiving anti‐myeloma chemotherapy in 2007‐2013. We matched BMA recipients (within 90 days of first chemotherapy) to nonrecipients using a propensity score, balancing patient‐, disease‐, and therapy‐related confounders. Cumulative incidence of skeletal‐related events (SREs) and overall survival (OS) was compared in proportional hazard models accounting for competing risks and immortal‐time bias.

**Results:**

Among 4611 patients with median age of 76 years, 51% received BMA. Bone‐modifying agents use remained steady over time (*P* = .87) and was significantly less frequent for patients who were older, with comorbidities, without prior SRE, and those treated without bortezomib or lenalidomide. In a propensity score‐matched cohort, BMA recipients experienced a lower incidence of SRE (11.0% vs 14.6% at 3 years; subhazard ratio, 0.73; 95% CI, 0.60‐0.89) and better OS (53.3% vs 47.8% at 3 years; hazard ratio, 0.86; 95% CI, 0.77‐0.95). The results were consistent in the subgroup (76%) treated with bortezomib and/or immunomodulatory drugs (IMiDs). The incidence of osteonecrosis of the jaw (ONJ) was 3.2% at 3 years.

**Conclusions:**

In this observational study, the observed benefits of early BMA administration among patients treated with contemporary anti‐myeloma regimens were similar to historical clinical trials. Frequent omission of BMA highlights a remediable deficiency in the quality of supportive care, and suggests that timely administration may be a useful indicator of quality care in myeloma.

## Graphical abstract

Despite the known survival benefits, only 51% of Medicare beneficiaries treated for myeloma received BMA as part of their first‐line chemotherapy. Survival advantage with early BMA administration in the era of bortezomib/lenalidomide therapy is similar to benefits observed in previously conducted clinical trials.

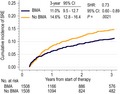

## 1 INTRODUCTION

Bone‐modifying agents (BMAs), which include intravenous bisphosphonates (zoledronate and pamidronate) and denosumab (a monoclonal antibody neutralizing the receptor activator of nuclear factor kappa‐Β ligand), are integral components of care for patients with plasma cell myeloma. In randomized clinical trials, addition of BMA to chemotherapy lowers the risk of myeloma‐related skeletal events and improves the quality of life, even in the absence of radiographically overt bone lesions.^1^, ^2^, ^3^, ^4^, ^5^, ^6^, ^7^ Furthermore, in the phase 3 Medical Research Council (MRC) Myeloma IX trial, administration of zoledronate (rather than oral clodronate) with anti‐myeloma therapy improved the overall survival (OS, with a hazard ratio [HR] of 0.86) and progression‐free survival (PFS, with HR of 0.89), suggesting a disease‐modifying effect.^5^, ^8^ Proposed mechanisms for this effect may involve BMA action on the bone microenvironment and/or direct cytotoxicity to malignant plasma cells.^9^, ^10^ As a result, multiple guidelines recommend administration of BMA to all patients initiating anti‐myeloma therapy who do not have prohibitive contraindications (eg, renal failure for bisphosphonates), but adherence to these recommendations in clinical practice is uncertain.^11^, ^12^, ^13^ It is also uncertain if survival benefit of BMA persists in the era of widespread use of bortezomib and lenalidomide as first‐line therapy, because the MRC Myeloma IX trial did not include these novel, highly active agents.

The benefits of BMA may be particularly important for older patients with myeloma, for whom debilitating skeletal‐related events (SREs) may potentially hamper effective anticancer therapy due to functional decline.^14^, ^15^ We hypothesized that early administration of BMA would be associated with better disease‐related outcomes among older patients, including those treated with bortezomib and/or lenalidomide. Our objective was to describe practice patterns with regard to the use of BMA in the United States (US), factors associated with omission of BMA, and to compare the risk of SRE and survival according to receipt of BMA as part of initial anti‐myeloma therapy. Prior studies have suggested significant disparities in the use of treatments for myeloma. Many novel anti‐myeloma agents are expensive, and socioeconomically disadvantaged groups may experience impaired access to them.^16^, ^17^, ^18^ Disparities in the application of supportive care have received less attention, yet BMA add to the cost and overall burden of an already complex therapy. Their use might thus constitute a valuable indicator of guideline adherence and overall quality of care in myeloma, important for policy‐makers and other stakeholders.

## 2 METHODS

### 2.1 Data source and study population

**Figure 1:**
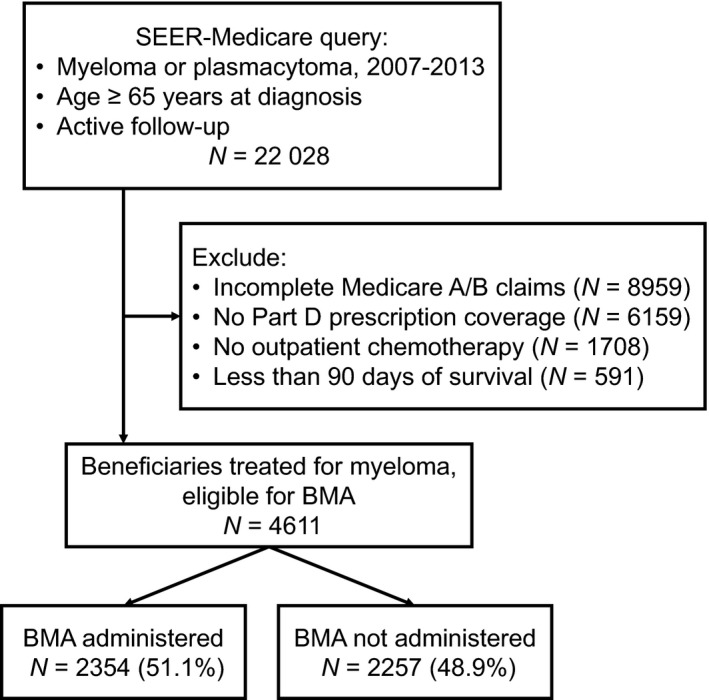
Cohort selection for analysis. BMA, bone‐modifying agent; SEER, Surveillance, Epidemiology, and End Results

This study was approved by the Institutional Review Board at Rhode Island Hospital. We used data from linked Surveillance, Epidemiology, and End Results (SEER)‐Medicare registry, which integrates cancer incidence data from 19 geographic areas in the US (covering approximately 34.6% of the population) with administrative claims for all inpatient and outpatient health services delivered to Medicare beneficiaries.^19^ Medicare is a federal health insurance program provided to individuals older than 65 years or with a disability. Some components, including prescription coverage for immunomodulatory drugs (IMiDs), require voluntary enrollment and payment of premiums, but beneficiaries with low income qualify for a subsidy decreasing or eliminating premiums and copayments.^18^, ^20^ For this study, we selected Medicare enrollees diagnosed with myeloma in 2007‐2013 who had complete Medicare inpatient, outpatient, and prescription claims, and a record of active outpatient chemotherapy (Figure 1). We excluded enrollees in Medicare‐sponsored private plans, whose records were not available. To avoid immortal‐time bias in the survival analysis, we also excluded patients who died within 90 days of starting first‐line chemotherapy.

## 3 MEASURES

Using administration codes for zoledronate, pamidronate, and denosumab, we identified the date of first BMA administration (Table S1). Of note, denosumab was approved for SRE prevention in solid tumors in 2010, but only in 2018 for myeloma. We defined our main exposure as administration of BMA within 90 days from the start of anti‐myeloma chemotherapy. We allowed this timeframe to avoid misclassifying patients with renal complications who required a delay in the initiation of BMA, or who received their first BMA as inpatient for hypercalcemia. The cutoff was further supported by empiric data, as the median time from the start of chemotherapy to BMA administration was 27 days (interquartile range [IQR], 7‐69 days), 80% of all first administrations occurred within 90 days, and only additional 9% occurred 91‐180 days after starting first‐line therapy.

We studied two outcomes of myeloma therapy. The cumulative incidence function (CIF) of a SRE was defined as time from start of therapy to first fracture of vertebrae, hip, pelvis, femur, performance of kyphoplasty or vertebroplasty, or a hospital admission with spinal cord compression. These events were ascertained from diagnosis and procedure codes in Medicare claims using previously described algorithms differentiating incident from prevalent (old) fractures.^21^, ^22^ Overall survival was measured from the start of chemotherapy until death or administrative censoring on 31 December 2015. We additionally examined the CIF of osteonecrosis of the jaw (ONJ), a major toxicity associated with BMA.

Clinical experts identified patient‐, myeloma‐, and treatment‐related covariates which might influence receipt of BMA or outcomes (Figure S1). These covariates included patients' age, sex, race, receipt of low‐income subsidies,^20^ performance status (using a validated claims‐based indicator),^23^ and the NCI comorbidity index.^24^ Although specific components of the Revised International Staging System (R‐ISS) for myeloma were not recorded in the data,^25^ we used claims‐based proxies of disease severity, based on health care services received during 12 months before chemotherapy, as previously described.^18^, ^20^ These included: initial histology (plasmacytoma or myeloma), anemia, neuropathy, chronic kidney disease and/or end‐stage renal disease, prior hospitalization, hypercalcemia, use of radiation therapy, history of osteoporosis or oral bisphosphonate use, and the presence of monoclonal gammopathy before diagnosis. We confirmed that our claims‐based prognostic index provided a similar or better discrimination of OS compared with the R‐ISS, distinguishing groups with 3‐year OS ranging from 19.7% to 70.1% (Figure S2). Treatment‐related covariates included diagnosis‐to‐treatment interval, treatment in a hospital outpatient or private physician's office, and specific first‐line regimen, classified as bortezomib ± cytotoxic chemotherapy, IMiD, IMiD plus bortezomib, corticosteroids only, and other.

### 3.1 Statistical analysis

Categorical variables were tabulated, and continuous variables were described as median and IQR. The relative risk of not receiving BMA was studied in a multivariable robust Poisson model, which included all prespecified covariates, regardless of statistical significance.^26^ We included a random intercept to account for practice variation according to each treating physician.

The average effect of BMA administration on outcomes (SRE, OS, and ONJ) was examined using a two‐stage propensity score method.^27^, ^28^ In the first stage, we estimated the propensity for BMA administration. We then matched BMA recipients with nonrecipients in a 1:1 ratio (without replacement, using a caliper of 0.2 times the standard error of the propensity score), thus generating two groups balanced with regard to the distribution of included covariates. We excluded subjects with extreme values of the propensity score outside of the overlap between the arms. Balance of confounders was confirmed using standardized differences of means. In the second stage, the effect of BMA was estimated in univariate outcome models. We used clustered standard errors to account for matched cohort, and censored observations after 3 years from the start of chemotherapy to eliminate the impact of later events unrelated to initial BMA administration. We used a proportional hazard model for OS and a competing‐risk model for CIF of SRE/ONJ, including death as a competing event.^29^ Because we could not ascertain if patients treated with steroids only received them for the myeloma indication, we repeated the analysis for patients treated with first‐line bortezomib and/or IMiDs. We assessed stability of results by progressively trimming the tails of the propensity score distribution and sensitivity to unobserved confounding by simulating an additional hypothetical confounder.^30^ We additionally examined the association between BMA administration (at any point after starting anti‐myeloma therapy) and OS in an extended (time‐split) model which assigns time‐at‐risk for every patient before receipt of BMA to the “untreated” group. All analyses were conducted using SAS 9.4 (SAS Institute Inc) and Stata 15.1/MP (StataCorp LP), with two‐sided *P* < .05 to determine statistical significance.

## 4 RESULTS

We identified 4611 eligible patients with median age of 76 years (IQR, 71‐81; Table 1), who started first‐line anti‐myeloma chemotherapy at median 1.2 months (IQR, 0.7‐2.1) from diagnosis. About 76% of patients received one of the novel agents: bortezomib (46%) or IMiD (43%, of which 75% was lenalidomide). BMA were administered to 2354 (51%) patients within 90 days from first chemotherapy, with median 5 (IQR, 3‐6) doses within the first 6 months, and median 9 doses (IQR, 5‐11) within the first 12 months. The most common BMA was zoledronate (83%), followed by pamidronate (16%) and off‐label denosumab (<1%). The proportion of BMA recipients did not significantly change between 2007 (50.1%) and 2013 (51.5%, *P* for trend = .87), even as the proportion treated with bortezomib or IMiDs increased from 58% to 82%, respectively.

**Table 1:** Characteristics of patients, stratified by receipt of BMA within 90 days from the start of anti‐myeloma chemotherapy

	All patients	BMA	No BMA	*P*
N	4611	2354	2257	
Age group, N (%)				<.001
<70	987 (21.4)	562 (23.9)	425 (18.8)	
70‐74.9	1213 (26.3)	640 (27.2)	573 (25.4)	
75‐79.9	1062 (23.0)	543 (23.1)	519 (23.0)	
80‐84.9	798 (17.3)	378 (16.1)	420 (18.6)	
≥85	551 (11.9)	231 (9.8)	320 (14.2)	
Sex, N (%)				.23
Women	2285 (49.6)	1187 (50.4)	1098 (48.6)	
Men	2326 (50.4)	1167 (49.6)	1159 (51.4)	
Race, N (%)				<.001
White	3749 (81.3)	1981 (84.2)	1768 (78.3)	
Black	613 (13.3)	256 (10.9)	357 (15.8)	
Asian or other	249 (5.4)	117 (5.0)	132 (5.8)	
Low income subsidy, N (%)				<.001
No	3277 (71.1)	1755 (74.6)	1522 (67.4)	
Yes	1334 (28.9)	599 (25.4)	735 (32.6)	
Histology, N (%)				.011
Plasma cell myeloma	4397 (95.4)	2263 (96.1)	2134 (94.6)	
Plasmacytoma	214 (4.6)	91 (3.9)	123 (5.4)	
Comorbidity index, N (%)^a^				<.001
0	1591 (34.5)	987 (41.9)	604 (26.8)	
1‐2	1771 (38.4)	909 (38.6)	862 (38.2)	
3‐4	876 (19.0)	335 (14.2)	541 (24.0)	
≥5	373 (8.1)	123 (5.2)	250 (11.1)	
Myeloma severity indicators, N (%)^a^				
MGUS before diagnosis	329 (7.1)	127 (5.4)	202 (8.9)	<.001
Poor performance status	661 (14.3)	327 (13.9)	334 (14.8)	.38
Hospitalization	2511 (54.5)	1259 (53.5)	1252 (55.5)	.18
Anemia	2818 (61.1)	1344 (57.1)	1474 (65.3)	<.001
Neuropathy	243 (5.3)	98 (4.2)	145 (6.4)	<.001
Kidney disease	1335 (29.0)	466 (19.8)	869 (38.5)	<.001
Prior ESRD	119 (2.6)	25 (1.1)	94 (4.2)	<.001
Hypercalcemia	566 (12.3)	365 (15.5)	201 (8.9)	<.001
Prior SRE	649 (14.1)	447 (19.0)	202 (8.9)	<.001
Osteoporosis	808 (17.5)	501 (21.3)	307 (13.6)	<.001
Oral bisphosphonate	546 (11.8)	303 (12.9)	243 (10.8)	.027
Prior radiation therapy	437 (9.5)	294 (12.5)	143 (6.3)	<.001
First‐line regimen, N (%)				<.001
Bortezomib	1052 (22.8)	557 (23.7)	495 (21.9)	
Bortezomib + cytotoxic agent	449 (9.7)	257 (10.9)	192 (8.5)	
Bortezomib + IMiD	630 (13.7)	406 (17.2)	224 (9.9)	
IMiD	1334 (28.9)	738 (31.4)	596 (26.4)	
Steroids only	773 (16.8)	235 (10.0)	538 (23.8)	
Other	373 (8.1)	161 (6.8)	212 (9.4)	
Site of treatment, N (%)^b^				.006
Physician's office	4110 (89.1)	2127 (90.4)	1983 (87.9)	
Hospital outpatient	501 (10.9)	227 (9.6)	274 (12.1)	

Abbreviations: BMA, bone‐modifying agent; IMiD, immunomodulatory drug; MGUS, monoclonal gammopathy of unknown significance; NCI, National Cancer Institute; SRE, skeletal‐related event.

a Binary indicators based on Medicare claims within 1 year before the start of chemotherapy (except for MGUS, which was assessed within 1 year before myeloma diagnosis).

b Defined by recording of >75% of claims for anti‐myeloma agents.

In a multivariable model, nonreceipt of BMA was significantly more frequent among patients who were older or who had more comorbidities, plasmacytoma, anemia, or renal disease, and among those who were treated without bortezomib or IMiDs (Table 2). Recipients of BMA had more frequent strong clinical indications like a prior SRE, hypercalcemia, osteoporosis, or radiation therapy. We observed no significant association between receipt of BMA and sex, race, low‐income status, or prior prescription for an oral bisphosphonate. Omission of BMA was 20% more frequent in hospital outpatient compared with physician office setting. It was also weakly correlated within each prescribing physician (intraclass correlation in a logistic model, 16.6%; 95% CI, 12.0%‐22.3%), indicating that to a relatively small extent, the decision to use BMA was related to physician preference rather than patient‐related factors.

**Table 2:** Factors associated with nonreceipt of BMA among Medicare beneficiaries with myeloma in a multivariable model

Variable	Percent receiving BMA	Adjusted RR for nonreceipt of BMA	95% CI	*P*
Age, y				
<70	56.9	Reference		.037
70‐74.9	52.8	1.06	(0.97‐1.16)	
75‐79.9	51.1	1.07	(0.98‐1.18)	
80‐84.9	47.4	1.11	(1.01‐1.22)	
≥85	41.9	1.17	(1.06‐1.30)	
Sex				
Female	51.9	Reference		.81
Male	50.2	1.01	(0.95‐1.07)	
Race				
White	52.8	Reference		.10
Black	41.8	1.07	(0.99‐1.16)	
Asian/other	47.0	1.10	(0.99‐1.23)	
Low income subsidy	44.9	1.07	(1.00‐1.15)	.05
Plasmacytoma histology	42.5	1.22	(1.09‐1.37)	.0004
Comorbidity index^a^	c	1.05	(1.02‐1.08)	.0003
MGUS before diagnosis^a^	38.6	1.11	(1.02‐1.22)	.020
Poor performance status^a^	49.5	0.91	(0.84‐0.99)	.036
Hospitalization^a^	50.1	1.01	(0.94‐1.08)	.76
Anemia^a^	47.7	1.11	(1.03‐1.18)	.003
Neuropathy^a^	40.3	1.10	(0.99‐1.21)	0.08
Kidney disease^a^	34.9	1.25	(1.16‐1.36)	<.0001
History of ESRD	21.0	1.32	(1.20‐1.45)	<.0001
Hypercalcemia^a^	64.5	0.73	(0.65‐0.83)	<.0001
Prior SRE^a^	68.9	0.72	(0.64‐0.81)	<.0001
Osteoporosis^a^	62.0	0.86	(0.78‐0.95)	.003
Oral bisphosphonates^a^	55.5	0.96	(0.87‐1.06)	.44
Prior radiation therapy^a^	67.3	0.70	(0.61‐0.81)	<.0001
First‐line regimen				
Bortezomib	52.9	Reference		<.0001
Bortezomib + cytotoxic	57.2	0.99	(0.87‐1.11)	
Bortezomib + IMiD	64.4	0.85	(0.76‐0.97)	
IMiD	55.3	1.07	(0.97‐1.17)	
Steroids only	30.4	1.45	(1.33‐1.59)	
Other	43.2	1.28	(1.14‐1.43)	
Time to chemotherapy	c	1.02	(1.01‐1.02)	<.0001
Treatment in hospital setting^b^	45.3	1.20	(1.09‐1.33)	.0003

Abbreviations: BMA, bone‐modifying agent; CI, confidence interval; ESRD, end‐stage renal disease; IMiD, immunomodulatory drug; RR, relative risk; SRE, skeletal‐related event.

a Indicators based on Medicare claims within 1 year before the start of chemotherapy (except for MGUS, which was assessed within 1 year before myeloma diagnosis).

b Defined by >75% of anti‐myeloma agent administrations in a hospital outpatient department.

c Continuous variable; months from myeloma diagnosis to start of chemotherapy.

With median follow‐up of 4.6 years, median OS was 3.0 years (95% CI, 2.9‐3.2), and was better for patients who received bortezomib or IMiD (median 3.2 vs 2.5 years; Figure S3). OS estimate at 3 years was 50.5% (95% CI, 49.0‐52.0). There were 686 recorded SREs, with 3‐year CIF of 12.8% (95% CI, 11.9‐13.8), not different according to receipt of bortezomib or IMiDs. The most common SRE was vertebral fracture (42.1%), followed by hip fracture (23.3%), kyphoplasty (19.7%), spinal cord compression (8.5%), and pelvis fracture (6.4%). Osteonecrosis of the jaw occurred in 116 patients, more often among BMA recipients (4% vs 1%).

**Figure 2:**
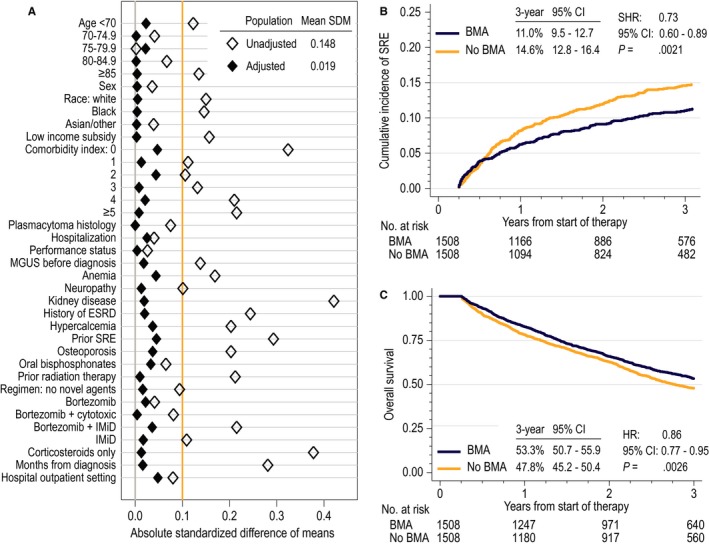
Outcome analysis: (A) balance of confounders after propensity score analysis, as determined by standardized differences of means (SDM); SDM of <0.1 conventionally indicates sufficient balance; (B) cumulative incidence function (CIF) of skeletal‐related events (SREs) in the propensity score‐matched cohort (N = 3016); outcome model reports subhazard ratio (SHR) with 95% confidence interval (CI); (C) overall survival in the propensity score‐matched cohort (N = 3016); outcome model reports hazard ratio (HR) with 95% CI. ESRD, end‐stage renal disease; IMiD, immunomodulatory drug

The propensity score analysis successfully balanced all included characteristics and resulted in 1508 matched pairs (total N = 3016) of patients who did or did not receive BMA. Standardized differences of means for all confounders were below 5%, indicating excellent reduction of bias from measured confounders (Figure 2A). The CIF of SRE was significantly lower for BMA recipients (11.0% vs 14.6% at 3 years; subhazard ratio [SHR], 0.73; 95% CI, 0.60‐0.89; Figure 2B). Overall survival was also better among BMA recipients (53.3% vs 47.8% at 3 years; HR, 0.86; 95% CI, 0.77‐0.95; Figure 2C). There was no differential effect according to the quantiles of the propensity score (*P* for interaction .87 for SRE, and 0.13 for OS), type of first‐line regimen (*P* = .14 and .54, respectively), or type of BMA received (*P* = .30 and .09, respectively). The risk of ONJ was significantly higher among BMA recipients (3.2% vs 0.8% at 3 years; SHR, 4.13; 95% CI, 2.19‐7.79). When the analysis was repeated in the subcohort of patients who received bortezomib and/or IMiDs (Figure S4), the results were consistent for all endpoints: SRE (SHR, 0.77; 95% CI, 0.61‐0.97), OS (HR, 0.87; 95% CI, 0.78‐0.98), and the risk of ONJ (SHR, 3.74; 95% CI, 1.88‐7.44).

Sensitivity analyses demonstrated stability of estimates when matching was conducted within cohorts with progressively narrower ranges of propensity score values, corresponding to patients with a more “average propensity” to receive BMA (Figure S5). We also found that the OS estimate was sensitive to unobserved confounding. In an adjusted model, an additional putative risk factor associated with a HR of 2.0 (eg, high‐risk cytogenetics in R‐ISS)25 would nullify the observed benefit of BMA if it were ≥10% more prevalent among BMA nonrecipients. The models for SRE were less sensitive, requiring at least 20% imbalance in such a factor between the arms to nullify the observed benefit. The analyses were not sensitive to the choice of ascertainment window for BMA administration between 60 and 120 days. The association between BMA receipt and OS was also consistently observed using the alternative time‐split extended Cox model (HR, 0.79; 95% CI, 0.73‐0.86).

## 5 DISCUSSION

In this population‐based study, we found that only about half of Medicare beneficiaries with myeloma treated with contemporary bortezomib‐ and lenalidomide‐based regimens received BMA with their initial chemotherapy. Lower risk of SRE and better OS among BMA recipients were quite similar in our study compared with prior randomized trials. These findings uncover a significant, remediable deficiency in the quality of care for patients with myeloma, and have significant implications for patients, clinicians, and other stakeholders interested in assessing the quality of care in oncology.

The 51% rate of BMA administration appears low, considering that the International Myeloma Working Group (IMWG) and the American Society of Clinical Oncology (ASCO) guidelines recommend BMA for all patients starting anti‐myeloma therapy.^11^, ^13^ However, we note that the IMWG guidance was published in 2013, and the 2007 ASCO statement recommended BMA for patients with lytic lesions or compression fractures.^31^ We could not ascertain results of radiographic studies in our population, but about 80% of myelomas present with lytic lesions at diagnosis.^11^ Furthermore, the presence of extensive bone disease would constitute an unfavorable risk factor for BMA recipients, so the benefits of treatment might be even higher than what we reported. Concurringly, the proportion of BMA recipients did not improve over time. Factors often discussed in the context of cancer disparities (sex, race, and socioeconomic status) did not significantly influence BMA use. Instead, omission of BMA was more frequent among patients receiving less effective anti‐myeloma regimens (without bortezomib or IMiDs), suggesting that optimal chemotherapy and supportive care are correlated. The use of all‐oral IMiD‐based regimens (like lenalidomide plus dexamethasone) was not associated with the omission of BMA, despite the requirement for additional parenteral injections. Because denosumab, unlike intravenous bisphosphonates, does not require adjustment for kidney function, most myeloma patients now have no contraindications to BMA. Bone‐modifying agent administration could thus be used as a measure of quality care for myeloma. Vitamin D deficiency, osteomalacia, and poor dental health remain potentially reversible contraindications. The 4% incidence of ONJ observed in our claims‐based analysis mirrors rates seen in clinical trials.^7^, ^8^, ^32^ Another recent study observed a low rate of BMA initiation in an academic practice (68%), which was substantially improved through a collaboration between the oncologists and clinical pharmacists.^33^ Interestingly, in our analysis, the omission of BMA was 20% more frequent in hospital‐based practices, suggesting that they might be a particular target for programmatic interventions to improve the quality of supportive care. However, the direct reasons why BMA was omitted from the initial therapy of nearly half of Medicare beneficiaries with myeloma cannot be confidently discerned from our claims‐based, retrospective analysis. Our observation calls for further qualitative research to understand them, and to address any potential barriers.

The effects of BMA on the risk of SRE (SHR, 0.73) and OS (HR, 0.86) were nearly identical to those observed in the prospective MRC Myeloma IX trial (0.76 and 0.86, respectively),^5^, ^8^ despite different populations and methods of SRE ascertainment. In that trial, participants were treated with cytotoxic chemotherapy regimens with or without thalidomide.^3^ Our study replicates the BMA advantage in the era when most patients receive novel, highly active anti‐myeloma agents (bortezomib or lenalidomide) and lower cumulative doses of dexamethasone compared with historical practice.^34^ Other trials, conducted largely before the widespread use of bortezomib and lenalidomide, have confirmed the lower risk of SRE with BMA, whereas survival benefits have not been seen consistently.^2^, ^4^, ^6^, ^35^, ^36^ Similarly to a prior network meta‐analysis and a phase 3 trial, we did not observe differential efficacy of various BMAs, with the caveat that denosumab use was too rare to draw conclusions.^4^, ^7^ Early institution of BMA may be paramount to achieve benefits, as the risk of SRE peaks in the first 2 years from diagnosis and is associated with higher mortality.^14^, ^15^ Our observational study cannot explain the mechanisms of BMA impact on survival in myeloma, but it demonstrates the effect to be independent of concurrent use of proteasome inhibitors or lenalidomide.^37^ Our results underscore the need to administer BMA, even as the novel chemotherapeutic regimens achieve higher efficacy and improved safety in older patients with myeloma.

Our results suffer from limited generalizability, as we had to exclude beneficiaries enrolled in private health plans, thus skewing the cohort toward older, retired individuals less likely to undergo intensive therapy with consolidative transplantation. To identify IMiD use, we had to further limit patients to those with Part D prescription coverage (50%‐70% of Medicare enrollees), though we captured most socioeconomically deprived recipients of low income subsidies.^18^ Potential unobserved confounding cannot be fully overcome using claims‐based constructs to approximate clinical variables because of their uncertain sensitivity and specificity. To alleviate this concern, we have shown that our indicators adequately stratified survival in myeloma, and we conducted sensitivity analyses for unobserved confounding. We also note that BMA recipients might be expected to have more risk factors related to extensive bone disease. Our SRE endpoint did not include the use of radiation, because targets or purpose of radiation therapy are not recorded in Medicare claims. We could not confirm the effect of BMA on PFS seen in the MRC Myeloma IX trial, because progression of myeloma was not recorded in SEER‐Medicare data. Although our approach precluded the evaluation of duration of BMA therapy, doses, or frequency of administration, such variation has shown no differential efficacy compared with more intensive schedules.^38^, ^39^ Our analytic framework did not allow examination of the effects of BMA started later in the course of the disease.

In conclusion, the benefits of BMA for older myeloma patients with regard to the risk of SRE and survival persist in the era of bortezomib‐ and/or lenalidomide‐based first‐line therapy. Bone‐modifying agents are administered to only half of potentially eligible Medicare beneficiaries, indicating a significant need to improve the quality of their supportive care. Further research should assess the impact of IMWG and ASCO guidelines in the context of less frequent BMA administration schedules and low nephrotoxicity of denosumab. We suggest that various stakeholders may consider the use of BMA as an indicator of quality of care for myeloma.

## CONFLICT OF INTEREST

AJO reports research funding from Genentech, TG Therapeutics, and Spectrum Pharmaceuticals; PMB reports no conflict of interest; JLR reports consulting and honoraria from Teva, Celgene, and Alexion Pharmaceuticals.

## AUTHOR CONTRIBUTIONS

AJO designed research, performed research, analyzed data, and wrote the paper; PMB interpreted the data and wrote the paper; JLR interpreted the data and wrote the paper.

## Data Availability

The data that support the findings of this study are available on request from the corresponding author. The data are not publicly available due to privacy or ethical restrictions.
