# Supplementary material for: Use of bone‐modifying agents and clinical outcomes in older adults with multiple myeloma
Source: Cancer Med. 2019 Sep 30;8(16):6945–54. doi: 10.1002/cam4.2591 (PMC6853813; doi:10.1002/cam4.2591)
Supplement: Supplementary file 1 [file CAM4-8-6945-s001.pdf]

Data supplement

Use of bone-modifying agents and clinical outcomes in older adults with multiple myeloma

Authors: Adam J Olszewski, MD [1,2],
Peter M Barth, MD [1,2],
John L Reagan, MD [1,2]

Affiliations
[1] Alpert Medical School of Brown University, Providence, RI
[2] Division of Hematology-Oncology, Rhode Island Hospital, Providence, RI

Corresponding author:

Adam J. Olszewski, M.D.
Assistant Professor of Medicine, Alpert Medical School of Brown University
Address: Comprehensive Cancer Center at Rhode Island Hospital
593 Eddy St., Providence, RI, 02903
Phone: 401-444-5435
Fax: 401-444-4184
E-mail: adam_olszewski@brown.edu

Supplemental Figure S1. Causal model for analyses, including treatment (the use of BMA), endpoints (SRE and overall survival), and confounders, as selected by clinical experts in myeloma.

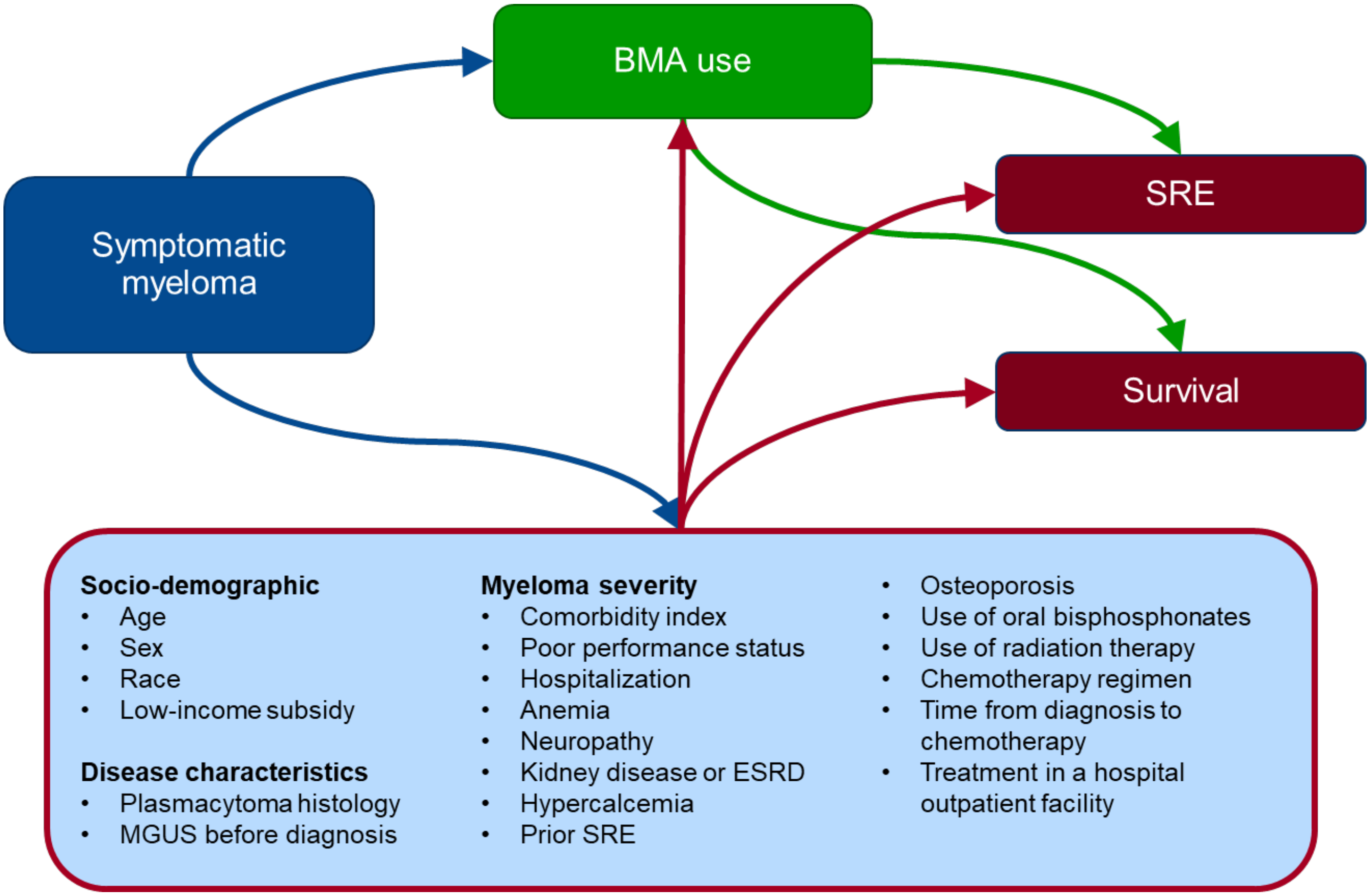

Supplemental Table S1. Definition of claims-based covariates in the study.

Variable	Timeframe	Definition
Low-income subsidy	At diagnosis	• As recorded in Part D denominator file for the month of myeloma diagnosis
MGUS	12 mo. before diagnosis	• ICD-9 code 273.*
Comorbidity index	12 mo. before chemotherapy	• NCI modification of Charlson Comorbidity Index [1]
Performance status	12 mo. before chemotherapy	• Davidoff Disability Indicator [2,3]
Hospitalization	12 mo. before chemotherapy	• Any short- or long-term inpatient admission claim
Anemia	12 mo. before chemotherapy	• ICD-9 code for anemia [a]<br>• Any red cell transfusion<br>• Any use of erythropoiesis-stimulating agent<br>• Any use of intravenous iron
Neuropathy	12 mo. before chemotherapy	• ICD-9 code for neuropathy or myopathy [a]
Kidney disease	12 mo. before chemotherapy	• Record of kidney disease on comorbidity Index[1]<br>• Visit with a nephrologist (according to Medicare provider/supplier specialty codes)
Hypercalcemia	12 mo. before chemotherapy	• ICD-9 code for hypercalcemia (275.42) [a]
Prior SRE	12 mo. before chemotherapy	• Fracture of vertebrae, hip, pelvis, or femur<br>• Any kyphoplasty or vertebroplasty<br>• Any spinal cord compression<br>• Ascertained according to previously published algorithms incorporating ICD-9 and HCPCS codes[4,5]
Osteoporosis	12 mo. before chemotherapy	• ICD-9 code 733.0* or 805.*
Use of oral bisphosphonates	12 mo. before chemotherapy	• Part D (outpatient prescription) claims for alendronate, ibandronate, or risendronate
Use of radiation therapy	12 mo. before chemotherapy	• ICD-9 or HCPCS code for radiation therapy administration in combination with a diagnostic ICD-9 code for myeloma or plasmacytoma (203.*)
Chemotherapy regimen	60 days from the start of chemotherapy	• Based on identification of specific anti-neoplastic drugs in HCPCS codes, Part D claims (for orally administered agents), NDC codes (in Durable Medical Equipment files), or ICD-9 codes for inpatient chemotherapy administration.<br>• Key agents identified included: bortezomib, lenalidomide, thalidomide, dexamethasone, prednisone, melphalan, cyclophosphamide, vincristine, and doxorubicin.
Time from diagnosis to chemotherapy	Measured in mo.	• Interval between diagnosis of myeloma (from the SEER registry) and first date of chemotherapy administration
Treatment setting	First 60 days of chemotherapy	• Based on the indicator of "place of service" in the Carrier or Hospital Outpatient files using all claims for chemotherapy administration.<br>• Outpatient prescriptions were treated as office-based setting.<br>• About 73% of patients had exclusively office-based claims.<br>• Hospital outpatient setting was empirically assigned if >75% of claims were recorded in that setting.
BMA administration	First 90 days from the start of chemotherapy	• HCPCS codes J2340, C9411 (pamidronate), J3847, J3488, J3489, Q2051 (zoledronate), and J0897 (denosumab)
Osteonecrosis of the jaw	Any time after the 90 days from chemotherapy	• Any occurrence of ICD-9 codes 733.45, 526.4, 526.89, 526.9

BMA: bone-modifying agents; HCPCS: Healthcare Common Procedure Coding System; ICD-9: International Classification of Diseases, 9th revision; NDC: National Drug Code; SEER: Surveillance, Epidemiology, and End Results

[a] to improve specificity, diagnostic codes had to be recorded at least once in inpatient claims, or at least twice, at least 30 days apart, in outpatient claims

Supplemental Figure S2. Evaluation of survival in myeloma using variables derived from cancer registry and Medicare claims.

The prognostic index was derived from a Cox model including all listed variables. Based on the model, patients were grouped into 4 quartiles according to the calculated risk. The resulting risk quartiles were evaluated in a univariate model. The Harrel's *c* concordance measure of this model was 0.65.

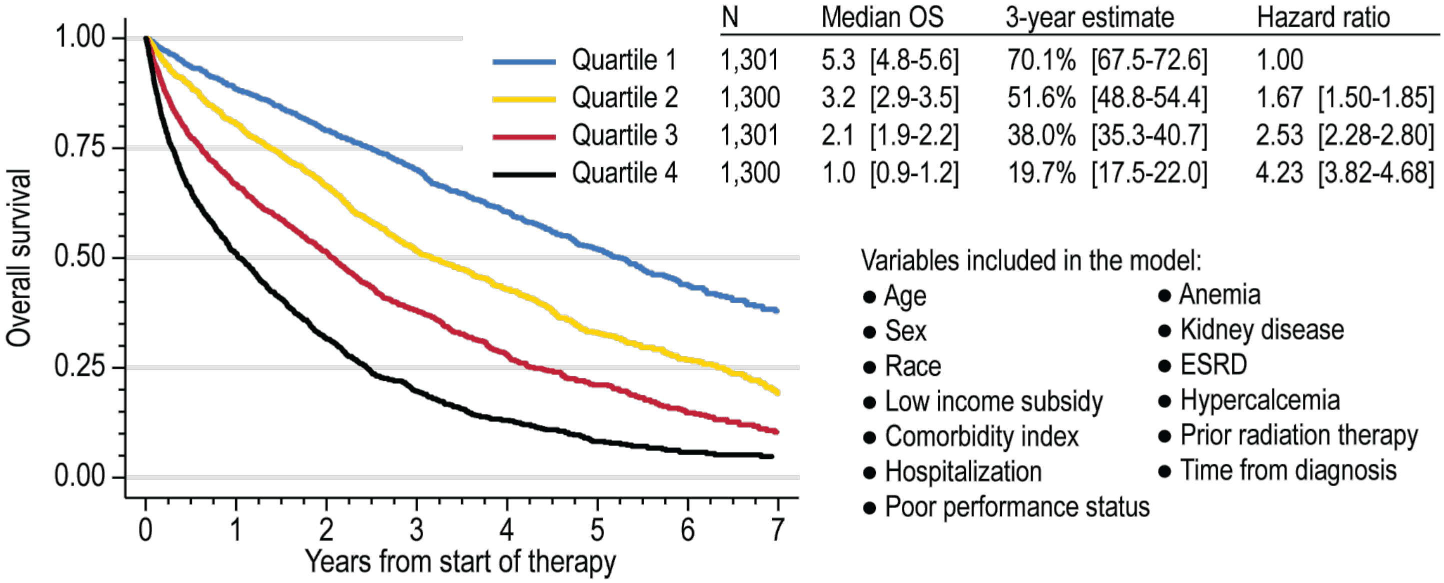

Supplemental Figure S3. Overall survival (A) and cumulative incidence function (B) of skeletal-related events (SRE) in the entire analytic cohort, stratified by receipt of bortezomib or IMiDs as part of first-line regimen.

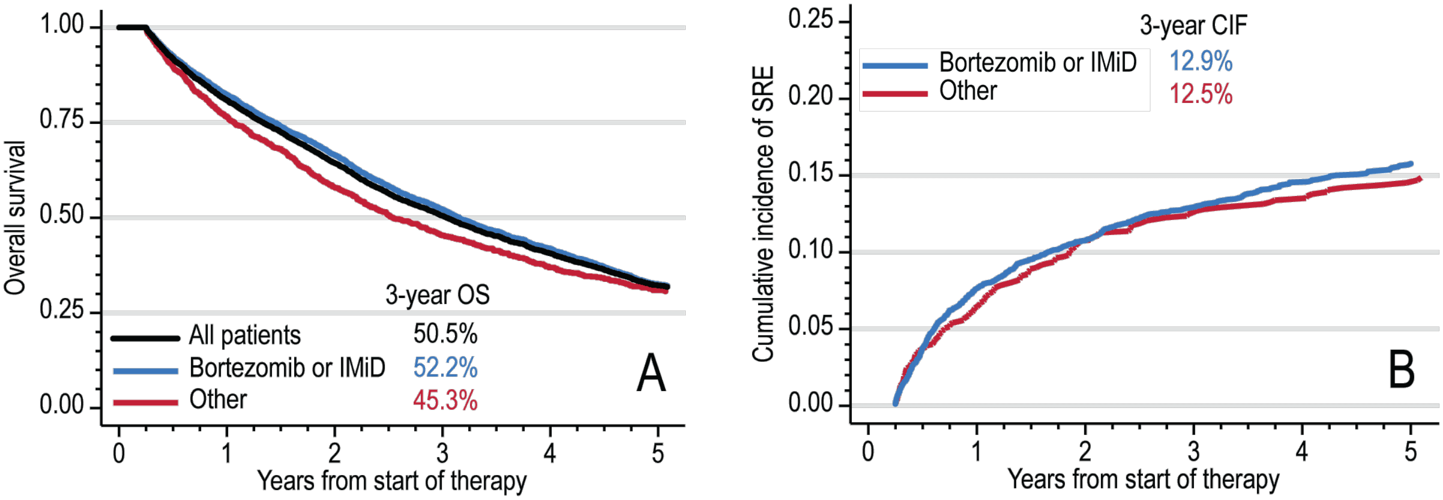

Supplemental Figure S4. Propensity score analysis in the subcohort of patients who received bortezomib and/or an IMiD as part of their first-line regimen for myeloma.

(A) balance of confounders, as determined by standardized differences of means (SDM); SDM of <0.1 conventionally indicates sufficient balance; (B) cumulative incidence function (CIF) of skeletal-related events (SRE) in the propensity score-matched cohort (*N*=2,316); outcome model reports subhazard ratio (SHR) with 95% confidence interval (CI); (C) overall survival in the propensity score-matched cohort (*N*=2,316); outcome model reports hazard ratio (HR) with 95%CI.

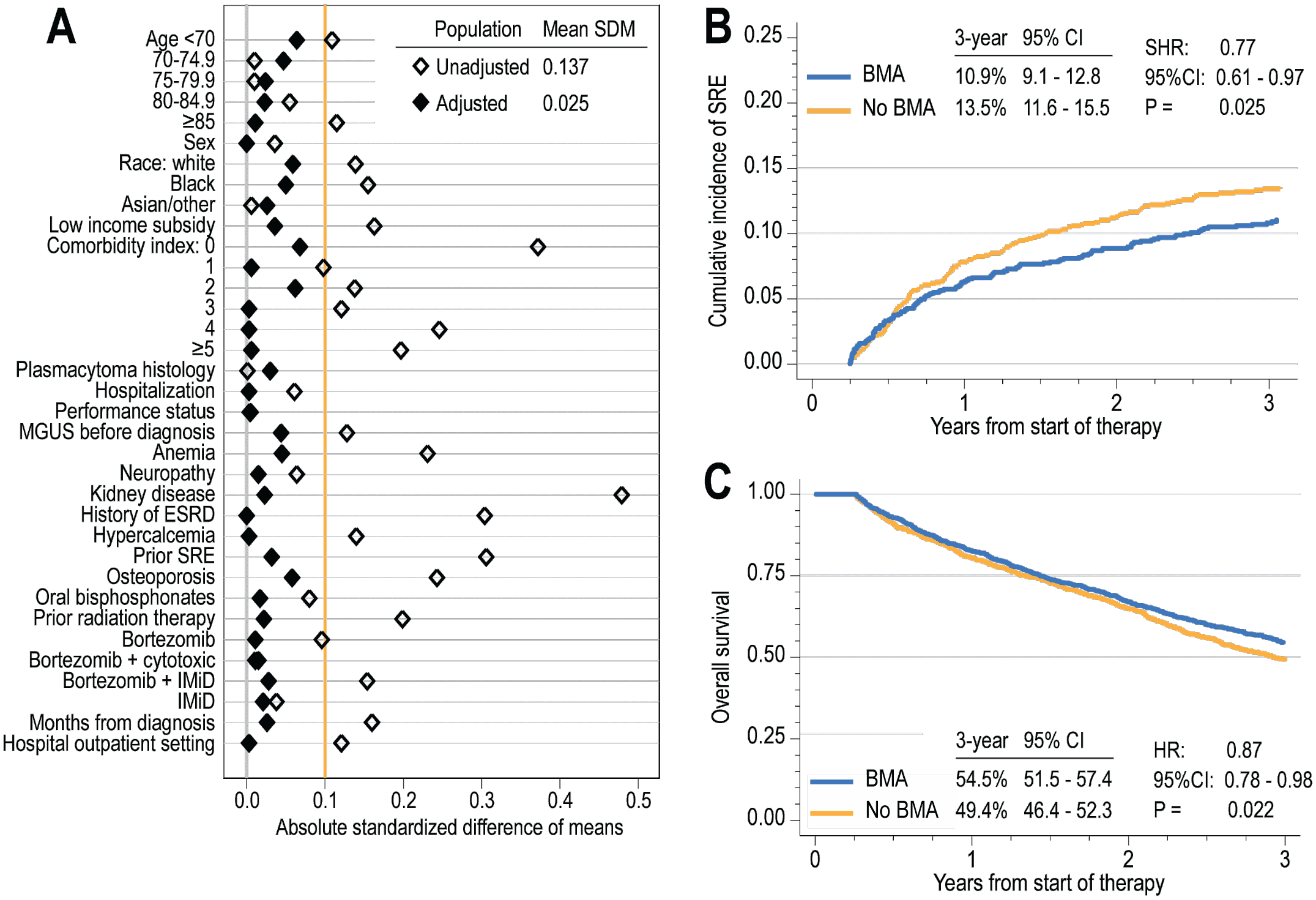

Supplemental Figure S5. Sensitivity analyses.

(A) estimate of subhazard ratio for the CIF of SRE, with 95% confidence interval (CI), after propensity score (PS) matching was conducted in subcohorts defined by progressively narrower ranges of allowed PS values; the estimate at 0% trim reproduces the main study result; as more % is trimmed, the number of matched pairs decreases, resulting in wider CI and increased mean bias; there was no evidence of a significant change in the estimate with higher trims; (B) OS estimate after analogous procedure; (C) a matrix showing estimates of subhazard ratio for the CIF of SRE, adjusted for an additional, unobserved, binary confounder, according to Lin et al.;[6] the confounder is simulated to have a hazard ratio of 2.0 (e^{γ}); if $\boldsymbol{\beta}^*$ is the baseline regression coefficient in the absence of the confounder, and $\boldsymbol{P_0}$ and $\boldsymbol{P_1}$ denote prevalence of the confounder among treated and untreated, then the adjusted coefficient ($\boldsymbol{\beta}$) is given by the equation: $\boldsymbol{\beta} \approx \boldsymbol{\beta}^* - \log\frac{e^{\gamma} * P_1 + (1-P_1)}{e^{\gamma} * P_0 + (1-P_0)}$; the 95% CI around the adjusted coefficient was calculated using the delta method; blue squares indicate statistically significant results; panels (E), (F), (G), and (H) show analogous analyses conducted in the subpopulation of patients receiving a novel agent (bortezomib or IMiD) as part of their first-line regimen.

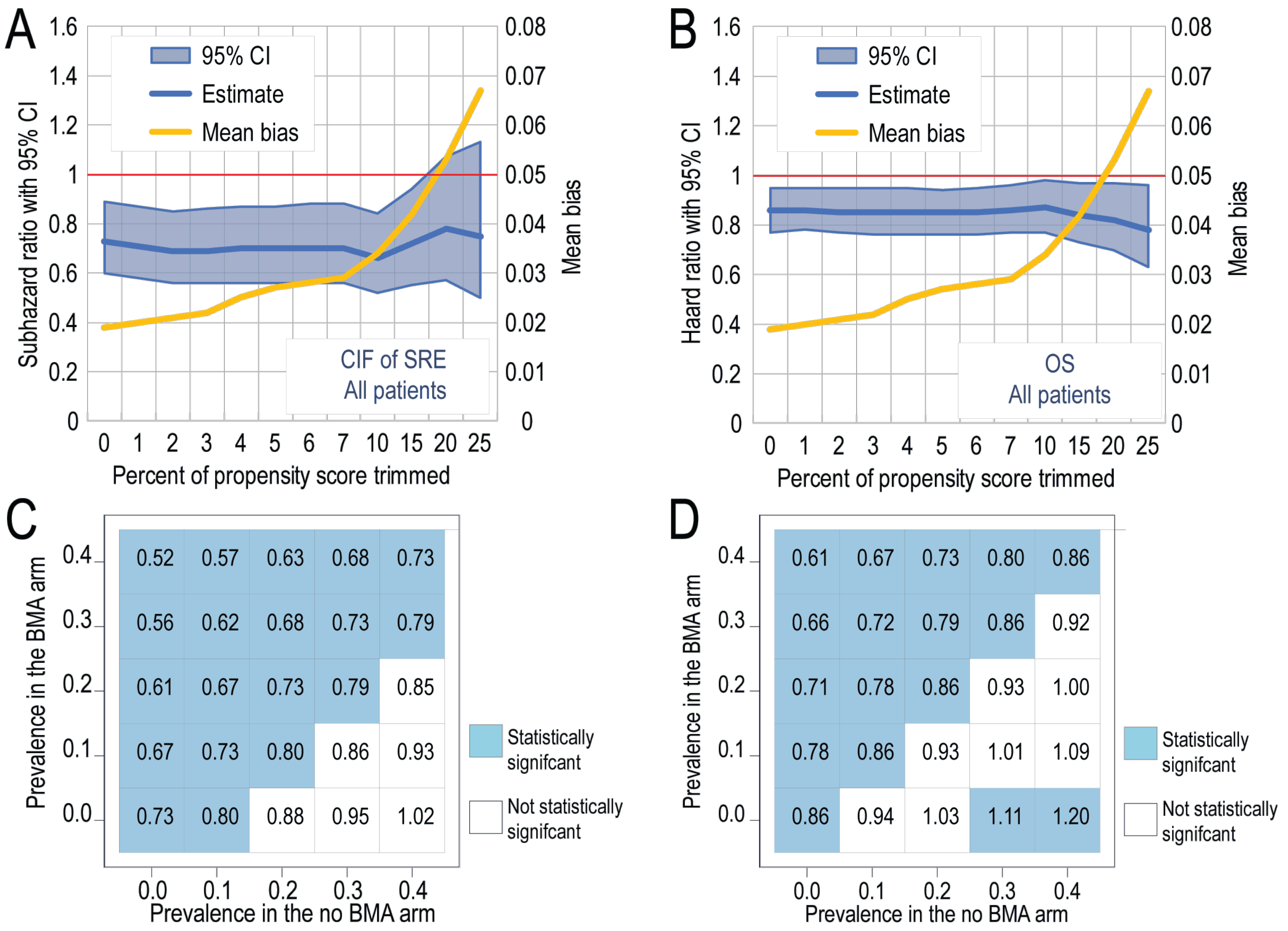

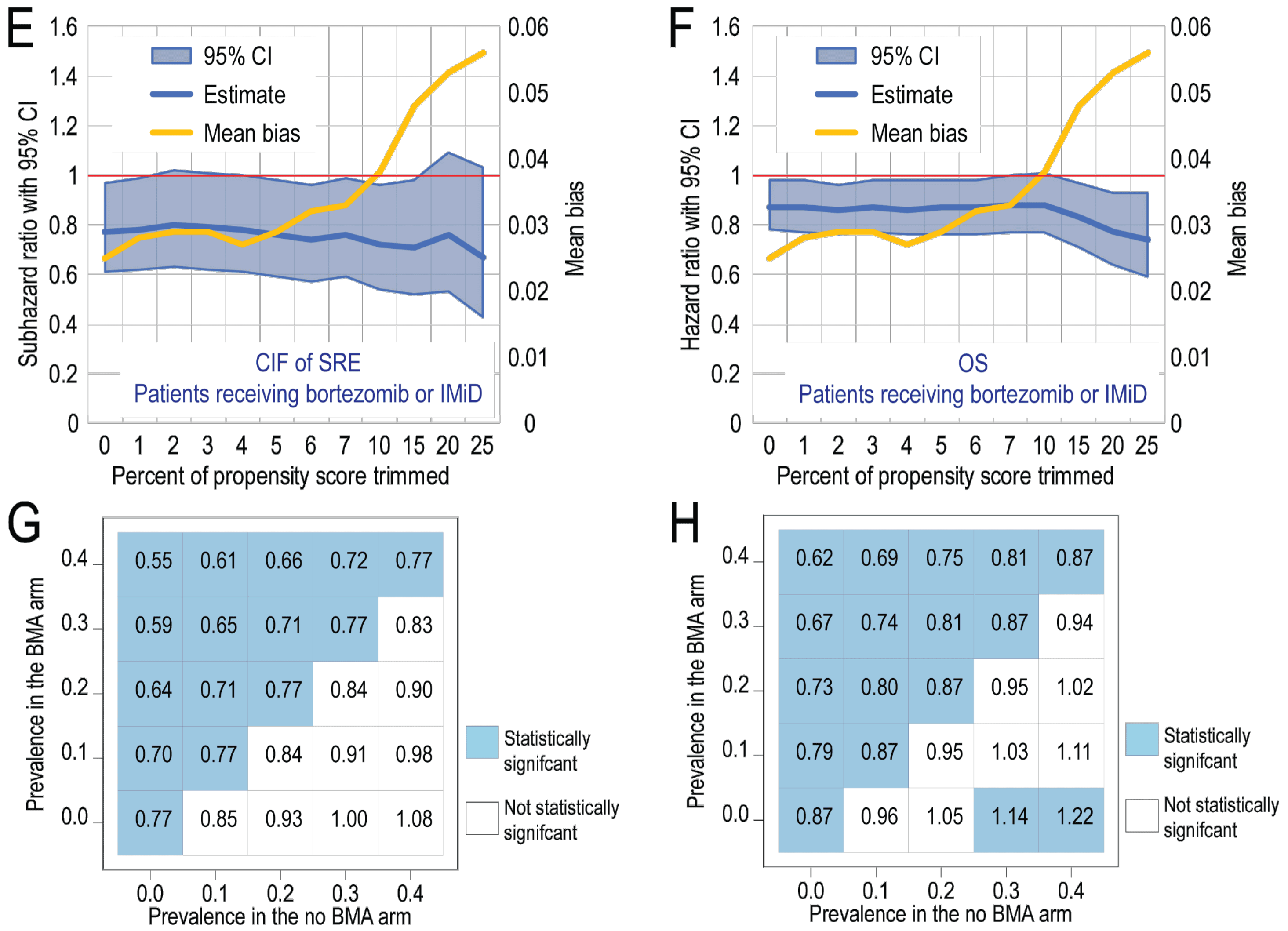
E
F
95% CI
Estimate
Mean bias
Subhazard ratio with 95% CI
Hazard ratio with 95% CI
Mean bias
CIF of SRE
Patients receiving bortezomib or IMiD
OS
Patients receiving bortezomib or IMiD
Percent of propensity score trimmed
G
H
Prevalence in the BMA arm
Prevalence in the no BMA arm
0.55 0.61 0.66 0.72 0.77
0.59 0.65 0.71 0.77 0.83
0.64 0.71 0.77 0.84 0.90
0.70 0.77 0.84 0.91 0.98
0.77 0.85 0.93 1.00 1.08
0.62 0.69 0.75 0.81 0.87
0.67 0.74 0.81 0.87 0.94
0.73 0.80 0.87 0.95 1.02
0.79 0.87 0.95 1.03 1.11
0.87 0.96 1.05 1.14 1.22
Statistically signifcant
Not statistically signifcant

Supplemental Table S2. Sensitivity analysis for the time window of BMA administration from the start of first-line therapy. The results for all endpoints were consistent when the window was varied between 60 and 120 days from the start of chemotherapy.

**Window for BMA receipt** (days from start of first-line chemotherapy)	**% of BMA recipients**	**Risk of SRE**		**OS**		**Risk of ONJ**	
		**SHR**	**95%CI**	**HR**	**95%CI**	**SHR**	**95%CI**
**All patients**							
**60**	72%	0.75	0.61-0.91	0.85	0.77-0.94	3.09	1.77-5.41
**90 (main analysis)**	80%	0.73	0.60-0.89	0.86	0.77-0.95	4.13	2.19-7.79
**120**	89%	0.69	0.55-0.86	0.87	0.79-0.97	4.92	2.29-10.57
**Patients receiving IMiDs and/or bortezomib**							
**60**	72%	0.82	0.66-1.03	0.84	0.75-0.94	2.58	1.44-4.63
**90 (main analysis)**	80%	0.77	0.61-0.97	0.87	0.78-0.98	3.74	1.88-7.44
**120**	89%	0.68	0.53-0.88	0.84	0.75-0.95	4.19	1.83-9.62

References

1. Klabunde CN, Potosky AL, Legler JM, et al: Development of a comorbidity index using physician claims data. J Clin Epidemiol 53:1258-1267, 2000

2. Davidoff AJ, Gardner LD, Zuckerman IH, et al: Validation of disability status, a claims-based measure of functional status for cancer treatment and outcomes studies. Med Care 52:500-510, 2014

3. Davidoff AJ, Zuckerman IH, Pandya N, et al: A novel approach to improve health status measurement in observational claims-based studies of cancer treatment and outcomes. J Geriatr Oncol 4:157-165, 2013

4. Taylor AJ, Gary LC, Arora T, et al: Clinical and demographic factors associated with fractures among older Americans. Osteoporos Int 22:1263-1274, 2011

5. Olszewski AJ, Gutman R, Eaton CB: Increased risk of axial fractures in patients with untreated chronic lymphocytic leukemia: a population-based analysis. Haematologica 101:e488-e491, 2016

6. Lin DY, Psaty BM, Kronmal RA: Assessing the sensitivity of regression results to unmeasured confounders in observational studies. Biometrics 54:948-963, 1998
